# Toxicity and Organ Distribution of Mercury in Freshwater Fish (*Oreochromis niloticus*) after Exposure to Water Contaminated Mercury (HgII)

**DOI:** 10.3390/toxics7040058

**Published:** 2019-11-19

**Authors:** Suhendrayatna Suhendrayatna, Nasrul Arahman, Luky Wahyu Sipahutar, Rinidar Rinidar, Elvitriana Elvitriana

**Affiliations:** 1 Chemical Engineering Department, Universitas Syiah Kuala, 23111 Banda Aceh, Indonesia; 2 Graduate School of Environmental Management, Universitas Syiah Kuala, 23111 Banda Aceh, Indonesia; nasrular@unsyiah.ac.id; 3 Animal Science Department, University of Muhammadiyah Tapanuli Selatan, 22716 Padang Sidempuan, Indonesia; luky.wahyu@um-tapsel.ac.id; 4 Pharmacology and Toxicology Department, Universitas Syiah Kuala, 23111 Banda Aceh, Indonesia; rinidar@unsyiah.ac.id; 5 Environmental Engineering Department, University of Serambi Mekkah, 23249 Banda Aceh, Indonesia; elvitriana@serambimekkah.ac.id

**Keywords:** exposure, accumulation, histopathology, mercury, LC_50_

## Abstract

The purpose of this study was to investigate the toxicity and the distribution of mercury (Hg) in the main tissues of freshwater fish (*Oreochromis niloticus*) after being exposed to water containing Hg(II). A sample group of 10 fish, of mean weight 80–100 g wet weight, were exposed to different concentrations of Hg (0.0012; 0.0049; 0.0141; 0.0524; 0.1126; and 0.5110 mg-HgII/L) for 72 h under controlled conditions using the static method in ponds. A control medium was also prepared in two replications. Mortality of fish was closely monitored, and the test was repeated three times. For the toxicity test, observations were based on behavior, mortality, and anatomical pathology. The methodology was based on the OECD guidelines for testing of chemicals and lethal concentration (LC_50_) and particularly using the probit method. Thus, the mean value was obtained from two replications and then further calculated by a software (MiniTab® 16 version). Prior to analysis, samples were first lyophilized. The total concentration of Hg accumulation in the fish organs was analyzed using heat-vaporization atomic absorption spectrometry (HV-AAS) and a MA2000 automatic mercury analyzer. Results showed that toxicity (LC_50_) of freshwater fish was 0.1435 mg-Hg(II)/L. The internal organs showed some pathological changes including pale gills, anemic eyes, and a whitish body color after the exposure. Furthermore, histopathologically, exposure to mercury might also affect other organs, such as gills, liver, and hepatopancreas. Mercury was found in trace amounts, and its accumulation was found to be at least in the gills. Meanwhile, the highest accumulation was found in the muscle tissue with approximately 5.7183 µg/g dry weight. If they are put in order, the mercury accumulation in the tissue organs was varied from the highest to lowest one: Muscle > eye > bone > head > gill. Finally, it can be concluded that the Hg exposure could affect the histopathological condition of the tested fish.

## 1. Introduction

Due to its toxicity, mercury became an international public concern, and it occurs in various forms and bioaccumulates in food webs where it can exert toxic impacts on the organism itself and its prey. Moreover, in some settings, inorganic mercury can be in high concentrations (e.g., some mines including gold artisan mines) and there is a need to know about the toxicity of inorganic mercury to local environments and fish species. The main purposes of this experiment were to reveal the acute toxicity and chronic histopathological effects of Hg(II) on the local fish *Oreochromis niloticus*. We found that the LC_50_ of fish for Hg (II) was 0.1435 mg-Hg(II)/L. After exposure to Hg(II), the pathological changes that occurred included pale gills, anemic eyes, and a whitish body color. Our findings confirm that histopathologically, internal organs of gills, liver, and hepatopancreas were greatly affected due to Hg(II) exposure.

The inflow of mercury (Hg) into an aquatic ecosystem occurs naturally as a result of mineral deposits, forest fires, volcanoes, oceanic emission, and crust degassing. The metal can also be released into ecosystems by human activities, such as smelters processing sulfide ores (i.e., in the production of metals such as iron, gold, copper, zinc, and lead), and other industrial activities, such as coal burning [1,2,3]. Because of its considerable potential hazard to public health and the environment, Hg pollution has become an international public health concern, and many studies have reported the presence of Hg in the environment [4,5,6]

The contamination of Hg compounds in marine environments is due to a natural phenomenon relating to anthropogenic discharge that contributes to the Hg flux increase and the alteration of chemical forms. Species of these altered forms may consequently elevate Hg levels in aquatic biota [7,8,9]. An increase in methylation rates will also cause this effect. Ruelas-Inzunza et al., 2004, observed that when elemental Hg (Hg0) is released to the atmosphere, it transforms into a soluble species (HgII) [10]. This mechanism is a transition from an inorganic compound into an organic form (mainly as methyl Hg), which is the first step in the accumulation process in aquatic biota. This form of Hg conversion can be increased by bacteria that occur under anaerobic and aerobic conditions. After bacterial accumulation and transformation to a neurotoxin, methyl Hg flows through food chains by biomagnification, and it eventually accumulates in other organisms and even humans [11].

Fish are one of the most important organisms in the aquatic food chain ecosystem. They have the ability to accumulate Hg at 1000 times higher in concentration than their surrounding aquatic environment [12]. Large-size marine organisms, such as dolphin and tuna, usually contain high levels of Hg. For medium-size fish, the Hg level is relatively small in their muscles. However, their internal organs, such as the liver, may contain much higher levels of Hg [13]. 

Ruelas-Inzunza, et al., 2004, reported that the distribution and relative concentrations of Hg in the main tissues of penaeid shrimps mostly accumulated in the hepatopancreas, followed by the muscle, then exoskeleton [10]. Limbong et al., 2003, reported that there was an increase in emissions of Hg from artisanal gold mining along the watershed of the three main rivers in North Sulawesi [14]. In Indonesia, the contamination of the rivers with mercury is mostly caused by such mining activities and affects areas like Tawalaan, Bailang, and Kima River in North Sulawesi; Barito, Kahayangan [14], Kr. Sabe River in Meulaboh, Aceh [15]; Kr. Sikulat River in Sawang, Aceh [16]; Wamsait River; and Kayeli Bay [17]. Almost all gold ore from mining is treated with a direct amalgamation procedure and produces a low gold concentrate, although a relatively high Hg concentration was released to the river. Inadequate technical knowledge and lack of regulation are mostly the major problems in all related gold mining processes occurring in Indonesia [14,17].

Human exposure to Hg is largely from the consumption of fish and products from the application of aquaculture. The toxicity risk to ecosystems from contamination of Hg have been increasing and have majorly affected fish toxicity. Thus, it is important to monitor it. However, the extent of toxicity and distribution of Hg in fish organs are poorly understood. This includes its histopathological changes in fish living in the Hg-contaminated tropics and sub-tropics.

Most of the studies on fish exposure to Hg investigated the kinetics of its uptake [18], and there are no available data on its effects on biochemical, survival, and heavy metal accumulation in Indonesian tropical fish species. Additionally, the histopathological examination has been increasingly recognized as a valuable tool for field assessment in its regard to the impact of environmental pollutants on fish [19]. Specific lesions, that occur when the organs of a fish are exposed to toxic substances under laboratory conditions, help to identify biomarkers of exposure. The purpose of this work is to understand the acute toxicity (LC_50_-72h) and chronic histopathological effects of Hg(II) on the freshwater fish *Oreochromis niloticus* after they were exposed to water contaminated with Hg(II). The aim of this experiment is to also improve our knowledge of the tissue and cellular mechanisms of Hg toxicity in fish and to analyze organ distribution of Hg. To determine organ distribution and accumulation of Hg in the main tissues of organisms, levels of Hg in the head, eye, gill, bone, and muscle of fish were measured.

## 2. Materials and Methods

### 2.1. Materials

The local freshwater fish species, *Oreochromis niloticus,* were applied as test organisms and obtained from a stock culture, Fish Seed Breeding and Farming Center, in Jantho, Aceh Besar District, Indonesia. All chemicals (such as HgCl_2_, NaOH, etc.) were obtained commercially from FUJIFILM Wako Pure Chemical Corporation. Activated alumina (additive B) and a mixture of sodium carbonate and calcium hydroxide (additive M) were purchased from Nippon Instruments Co. (NIC). DOLT3 dogfish liver, as a reference material (Hg 3370 µg/g), was obtained commercially from Canada National Research Council. The experimental equipment used were a Vacuum Oven Eyella VOS 450SD, glassware (PYREX®), muffle furnace, analytical balance (Sartorius), and a desiccator (Pyrex), and heat-vaporization atomic absorption spectrometry (HV-AAS) was performed with a MA2000 automatic mercury analyzer (Nippon Instruments Corporation).

### 2.2. Toxicity Test

Upon arrival at the laboratory, the fish were immediately allowed to be acclimatized in tap water at room temperature (30 ± 2 °C), with a pH range of 7.6–7.8. A condition of 12:12 light-to-dark cycle of at least two weeks before an in vivo initiation test was applied. Fish were fed twice a day with Hg-free algae dried powder. The toxicity test was conducted based on the OECD Guidelines for Testing of Chemicals (Lammer et al. 2009) [20]. Fish were exposed to different Hg concentrations for 72 h under controlled conditions using the static method in ponds. The six Hg concentrations used were 0.0012; 0.0049; 0.0141; 0.0524; 0.1126; and 0.5110 mg-Hg(II)/L. Hg-free controlled medium was also prepared with two replications for each. Ten fish (mean weight 80–100 g wet weight) were used for each designed concentration and mortality was thoroughly monitored, with the test being repeated three times. During the toxicity test, observations of the fish test sample were performed based on behavior, mortality, and anatomical pathology. Behavioral observations were carried out during the 72 h exposure period; each treatment group was observed periodically and recorded every 4 h. The behavioral indicators observed were restlessness, sudden jerks, swimming erratically, and lack of appetite. The preparation of the gills, liver, and hepatopancreas was done using a surgical instrument, in which afterwards the organs were processed into histopathological preparations. The changes were observed under the microscope and photographed using a special microscope.

### 2.3. Ethical Approval 

The protocols in this research had ethical approval No. 34/KEPH/I/2018 (date 16 January 2018) from the veterinary ethics committee of the Faculty of Veterinary Medicine, Syiah Kuala University, Banda Aceh, Indonesia. The maintenance and animal application for this research based on the standard method was chosen with the approval of the committee.

### 2.4. Mercury Accumulation Test

*Oreochromis niloticus* specimens were exposed to 30 L of dilute medium containing 0.5110 Hg(II) under static conditions for 72 h within the pH range of 7.6–7.8. The fish were fed daily with Hg-free algae dried powder, equivalent to approximately 2% of their body weight. A control medium (Hg-free) was also prepared for two replications. After three days of observation, organs from each organism (bone, head, gill, muscle, and eyes) were dissected from each individual fish, lyophilized (Vacuum Oven Eyella VOS 450SD at −60 °C using liquid nitrogen), and grained.

### 2.5. Mercury Analysis in Fish Tissue

The total Hg in the tissues was analyzed using a heat-vaporization atomic absorption spectrometry (HV-AAS) and a MA2000 automatic mercury analyzer (Nippon Instruments Co., NIC, Ltd.). Hg(II) standard solutions were adequately diluted and added directly to the ceramic sample boats. According to the manufacturer’s protocol, samples of the organisms (30–40 mg) were added to ceramic boats with two types of additives; one consisted of activated alumina (additive B) and the other was a mixture of sodium carbonate and calcium hydroxide (additive M). All ceramics boats and additives were heated in a muffle furnace at 700–800 °C for 6 h to remove any background Hg. The standard solutions and samples in the boats were placed in the sample loader part of the analyzer to measure the total Hg concentration in the samples. All glasses and ceramic boat wares were cleaned by soaking with a cleaning solution followed by a Milli-Q water rinse before use. Quality control was assured by DOLT3 dogfish liver (Canada National Research Council) with a certified value 3370 µg-Hg/g for total Hg. Our analytical result of total mercury level was 3720.82 µg-Hg/g, and the recoveries of Hg were around 110.4%.

### 2.6. Histopathology Preparations

Hg-exposed fish were dissected after 96 h to obtain fractions of gills, hepatopancreas, and livers. The gills were fixed in 10% Davidson solution, whereas the hepatopancreas and liver were fixed using 10% formalin solution. Furthermore, the two organs were prepared for histopathological examination by applying the Haematoxylin and Eosin staining technique. Histopathological sample pieces were viewed under a light microscope by applying alternate magnifications to obtain clear images.

### 2.7. Data Analysis

The concentration used was calculated by the lethal concentration (LC_50_) using the probit method. Further data analysis was supported with software MiniTab® 16 version 2010. Microscopic examination was performed by looking at the gill and liver histopathology images, and they were analyzed descriptively and qualitatively, based on the changes that occurred. All results were then compared with the control.

## 3. Results and Discussion

### 3.1. Toxicity of Oreochromis niloticus against Hg(II)

**Figure 1:**
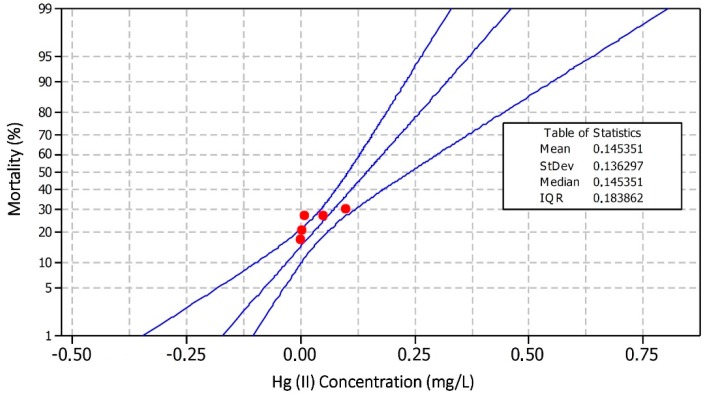
Linear regression probit model test, LC_50_ Hg concentration to mortality of freshwater fish, *Oreochromis niloticus* (probability plot for mortality, normal 95% CI, probit data ML estimation).

From this research, it was observed that fish mortality obtained during 72 h of the toxicity test was at varying levels, as exhibited on Table 1. The mortality in each Hg(II) concentration treatment was found to fluctuate (1.67; 2.0; 2.33; 2.67; 3.0; and 10 fish), while no mortality was found in the control treatment (no Hg in this treatment). The fish mortality data were then analysed, based on the probit method, to obtain a lethal concentration (LC_50_). The number of test fish mortality and LC_50_ values are presented in Table 1 and Figure 1, respectively.

The fish mortality ratio and Hg(II) concentration data were analyzed using the probit method, and analysis was supported by software MiniTab® 16 version. Acute mortality at LC_50_ for 72 h was found at 0.1453 mg-Hg(II)/L, equivalent to 0.4843 mg-Hg(II) in 30 L of water. This resulted in 50% mortality rate for the tested fish. The results of LC_50_ probit regression modeling showed that the high mortality rate of the tested fish was strongly influenced by the concentration level, given that (*p* < 0.05). Figure 1 shows the result of the probit regression model of the toxicity test, LC_50_-72 h of Hg(II) to *Oreochromis niloticus*. Even though the tested fish experienced different times of death, the mortality rate showed differences in the number of deaths that occurred in each group of the tested fish.

This result shows the mean value of 50% (*y*) mortality percentage. The regression line was at the point of 0.1453 in the concentration line (*x*), where the measured log concentration ratio of mortality from each concentration was shown at red point 0.0012; 0.0049; 0.0141; 0.0524; and 0.1126 mg-Hg(II)/L, respectively. From these values, it was determined that the Hg(II) concentration level correlated with the number of fish mortality percentage (*p* < 0.05). The higher the concentration of Hg(II) given, the higher the mortality of the tested fish. This result indicated the direct proportional relationship between mortality and concentration of mercury as a test chemical. The mercury toxicity was lower in freshwater fish, *Oreochromis niloticus*, compared with *Esox lucius*, which have a LC_50_ of 0.080 mg-Hg (II)/L [21].

### 3.2. Mercury Accumulation and Its Organ Distribution

The differential accumulation of Hg in various tissues in fish was observed. The smallest amount of accumulated Hg was found in the gills (trace amounts), whereas the maximum concentration was located in the muscle (5.7183 µg/g dry weight), as shown in Table 2. Hg concentrations were found to be varied in the following order from highest to lowest: Muscle > eye > bone > head > gill. The results indicate that Hg has different effects on various organs. The major parts of the total body that accumulated at different concentrations and at various exposure times were the liver, kidney, and gills [22]. Some researchers reported that concentrations of metals in the digestive tract of fish bodies inhabiting the natural water [23,24] were closely related to their dietary uptake route. The high accumulation of mercury in muscles was also reported by [25]. They reported the carnivorous fish, such as scorpion fish, sea bream, and Japanese whiting, tended to show higher Hg accumulation in the muscle. Hg level in muscle was significantly higher than the liver in Pacific saury and Japanese whiting. Furthermore, muscle tissue of predatory fishes contained significantly higher content of total mercury than muscle tissue of nonpredatory fishes [21]. In contrast, [26] found the concentration of mercury in tissues of six species of freshwater fish from the Kpong hydroelectric reservoir decreased in the order: Liver > muscle > intestine > stomach > gonad > gill > swim bladder. Mercury concentration in the tissues ranged from 0.005 to 0.022 μg/g wet weight.

Furthermore, not all tissues receive the same blood flow, and the distribution of Hg in the various tissues might be different as a result [27]. Hg accumulation in tissues is a function of the Hg intake as well as the clearance rates of the individual organs. The significant correlation between Hg concentrations and various tissues indicated that co-accumulation of Hg is occurring in tissues in a form that might be suitable to be gathered and stored there.

### 3.3. Pathology Conditions

**Figure 2:**
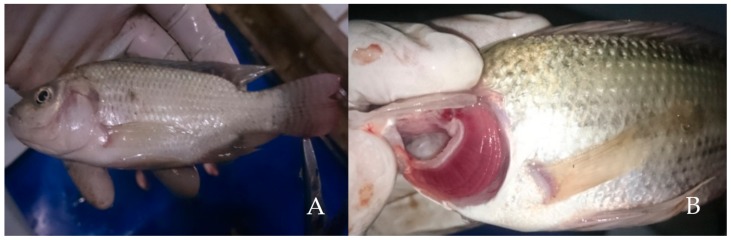
Pathological conditions of freshwater fish (*Oreochromis niloticus*) after exposure to Hg(II). (**A**) whitish body and eyes and (**B**) pale gills color.

Observations on behavior show that the test fish did not exhibit abnormal symptoms under toxicity test. However, dead fish showed special pathological conditions on the body including pale gills, anemic eyes, and whitish body color. The description of pathological anatomical changes among freshwater fish, *Oreochromis niloticus*, after exposure to Hg(II) is illustrated in Figure 2.

Furthermore, observations on anatomical pathology in the liver, spleen, and intestine showed that after exposure to Hg, the test fish did not show significant changes in pathological conditions. Fish behavior also did not experience observable changes after exposure to Hg. Based on these results, it can be interpreted that Hg toxicity in fish did not have a significant effect either on changes in fish behavior or on anatomical and pathological conditions of internal organs. However, the effect of Hg could influence changes in anatomical pathology of the body surface, which experienced a direct exposure, such as eyes, gills, and skin.

### 3.4. Overview of Histopathology

**Figure 3:**
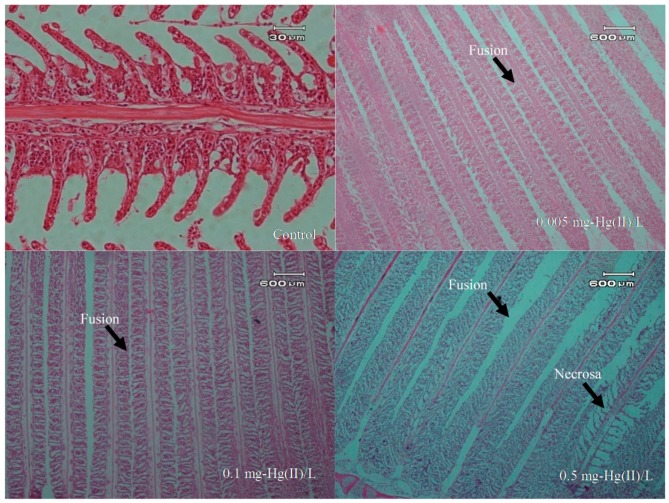
Photomicrograph description of freshwater fish (*Oreochromis niloticus*) after exposure to Hg(II) (20 × 10).

Histopathologically, there were several organ changes observed under microscope. The condition of the gill, liver, and hepatopancreas organs showed the influence of metal toxicity of Hg(II) in the water phase. Observations of histopathological gill, liver, and hepatopancreas organ damage are presented in Figure 3.

**Figure 4:**
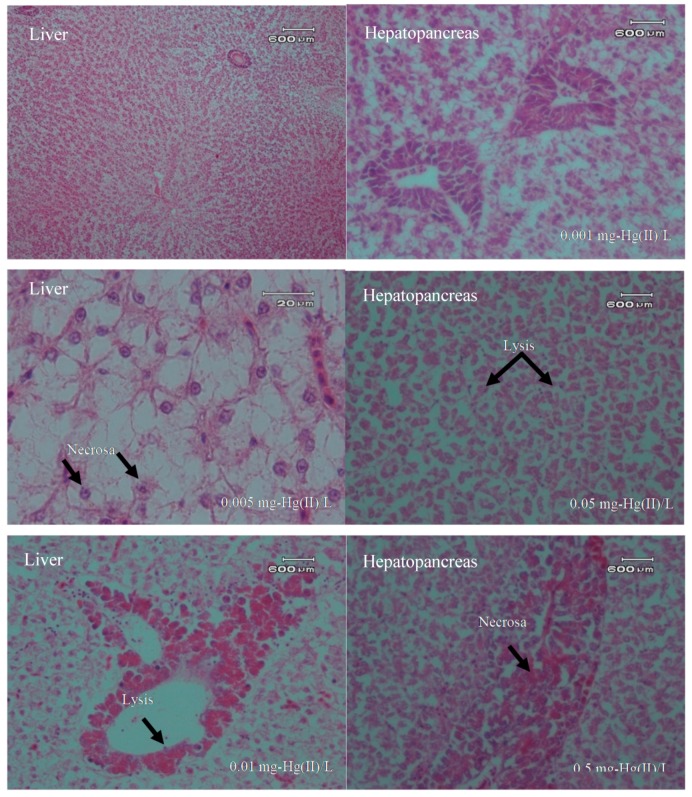
Description of photomicrograph of liver and hepatopancreas of freshwater fish (*Oreochromis niloticus*) after exposure to Hg(II) (20 × 10).

The images from the photomicrographs in Figure 3 and Figure 4 reveal some histopathological changes in the organs due to the exposure to Hg. All gill organs showed changes in lamella fusion for each treatment group as well as necrosis in more severe conditions. Microscopic images for the livers showed cell lysis occurred in the cytoplasm and liver hepatocytes, as well as necrosis. The hepatopancreas also showed the same conditions in which its cells experienced cell lysis and some necrosis. Histopathological observations of gill, liver, and hepatopancreas can be seen in Table 3.

## 4. Conclusions

The results show that the toxicity (LC_50_) of Hg(II) for the freshwater fish *Oreochromis niloticus* was found to be in the amount 0.1435 mg-Hg(II)/L. After exposure to Hg(II), fish showed pathological changes, namely pale gills, anemic eyes, and whitish body color. Histopathologically, exposure to Hg also affected the organs of the gills, liver, and hepatopancreas. The accumulation of Hg in fish was minimal in the gills, while accumulation was high in the muscle (5.7183 µg/g dry weight). Hg concentrations were varied in the order: Muscle > eye > bone > head > gill. It can be concluded that Hg exposure at concentrations of 0.0012; 0.0049; 0.0141; 0.0524; 0.1126; and 0.5110 mg-Hg(II)/L could affect the condition of the tested fish histopathologically.

## Figures and Tables

**Table 1:** Mortality of freshwater fish *(Oreochromis niloticus)* during 72 h toxicity test.

Hg Concentration in the Water Phase (mg/L)	Repetition	No. of Fish	Mortality	Average Mortality
0 (control)	1	10	0	0.0
	2	10	0	
	3	10	0	
0.0012	1	10	2	1.67
	2	10	1	
	3	10	2	
0.0049	1	10	2	2.0
	2	10	2	
	3	10	2	
0.0141	1	10	3	2.33
	2	10	2	
	3	10	2	
0.0524	1	10	3	2.67
	2	10	3	
	3	10	2	
0.1126	1	10	3	3.0
	2	10	3	
	3	10	3	
0.5110	1	10	10	10
	2	10	10	
	3	10	10	

**Table 2:** Accumulation and its organ distribution of Hg in freshwater fish (*Oreochromis niloticus*) after exposure to water contained high concentration of Hg(II).

Hg(II) in the Water Phase (mg-Hg)/L	Hg Concentration in An Organ (µg-Hg/g Dry Cells)				
	Head	Muscle	Eye	Bone	Gill
0 (Control)	0.26 ± 0.37 **	0.48 ± 0.22 **	0.37 ± 0.04 **	0.42 ± 0.04 **	0.35 ± 0.08 **
0.511	0.376	5.718	4.310	3.960	tr *

Note: * trace, ** average of data from two replicated series of measurements.

**Table 3:** The level of damage to gill, liver, and hepatopancreas organs after freshwater fish (*Oreochromis niloticus*) exposure to Hg(II).

Organs	Histopathology	Damage Level		
		1	2	3
Gill	Lamela fusion	+++	+++	+++
	Necrosa	−	−	++
Liver	Lysis	+++	++	+++
	Necrosa	+	+	+
Hepatopancreas	Lysis	++	++	+++
	Necrosa	+	+	++

Note: (−) none/not significant (normal); (+) damage less than 30% viewing area (light); (++) damage 30–70% viewing area (medium); (+++) damage more than 70% of viewing area (weight).
